# No serological evidence for neuronal damage or reactive gliosis in neuro-COVID-19 patients with long-term persistent headache

**DOI:** 10.1186/s42466-022-00217-5

**Published:** 2022-10-31

**Authors:** Laura de Boni, Alexandru Odainic, Natalie Gancarczyk, Luisa Kaluza, Christian P. Strassburg, Xenia A. K. Kersting, Joseph M. Johnson, Ullrich Wüllner, Susanne V. Schmidt, Jacob Nattermann, Gabor C. Petzold

**Affiliations:** 1 Institute of Aerospace Medicine, German Aerospace Center, Cologne, Germany; 2 Biomedical Center (BMZ 2), Institute of Innate Immunity, University Hospital Bonn, Bonn, Germany; 3 Division of Vascular Neurology, Department of Neurology, University Hospital Bonn, Bonn, Germany; 4 Department of Internal Medicine I, University Hospital Bonn, Bonn, Germany; 5 Department of Psychiatry and Psychotherapy, University Hospital Mainz, Mainz, Germany; 6 Quanterix Corporation, 900 Middlesex Turnpike, Billerica, MA 01821 USA; 7 German Center for Neurodegenerative Diseases (DZNE), Bonn, Germany; 8 Department of Neurodegenerative Diseases and Gerontopsychiatry, University Hospital Bonn, Bonn, Germany; 9 German Center for Infection Research (DZIF), University Hospital Bonn, Bonn, Germany; 10 Department of Microbiology and Immunology, The Peter Doherty Institute for Infection and Immunity, University of Melbourne, Melbourne, Australia

**Keywords:** Post-acute sequelae of SARS-CoV-2 infection, Post-COVID-19, Headache, NfL, GFAP, Biomarkers

## Abstract

Recent studies have indicated that long-term neurological sequelae after COVID-19 are not accompanied by an increase of canonical biomarkers of central nervous system injury in blood, but subgroup stratifications are lacking. This is a particular concern in chronic headache, which can be a leading symptom of Post-COVID diseases associated with neuronal damage such as vasculitis or autoimmune encephalitis. We here compared patients with mild Post-COVID-19 syndrome and persistent headache (persistent Post-COVID-19 headache) lasting longer than 12 weeks after the initial serological diagnosis, to patients with mild and severe COVID-19 and COVID-19-negative controls. Levels of neurofilament light chain and glial fibrillary astrocytic protein, i.e. markers of neuronal damage and reactive astrogliosis, were lower in blood from patients with persistent Post-COVID-19 headache compared to patients with severe COVID-19. Hence, our pilot serological study indicates that long-term Post-COVID-19 headache may not be a sign of underlying neuronal damage or neuroinflammation.

Neurological post-acute sequelae of SARS-CoV-2 infection (PASC) are common, although direct viral infection of the central nervous system (CNS) is rare [3, 6]. Instead, inflammatory mechanisms, parenchymal hypoxia or microvascular injuries may contribute to the development of CNS injury, raising the possibility that these long-term symptoms may be accompanied by systemic biomarkers of neuronal damage or neuroinflammation.

Accordingly, recent studies evaluated neurofilament light chain (NfL) as a marker of neuronal injury and glial fibrillary acidic protein (GFAP) as a marker of reactive astrogliosis and neuroinflammation in the blood of patients with acute COVID-19 and PASC. Although patients with severe acute COVID-19 had higher concentrations of NfL and GFAP than moderately/mildly affected COVID-19 patients or controls [4], the levels of these biomarkers subsequently returned to normal levels and were not correlated with persistent neurological symptoms in patients with PASC [4]. However, these initial studies did not allow a subgroup analysis according to chief neurological complaint or primary symptoms. This is particularly relevant for persistent headache, a common and debilitating PASC symptom for several reasons [5]. First, headache was a leading symptom associated with increased NfL and GFAP levels and increased mortality in acute COVID-19 patients [1]. Second, new persistent headache may also be an initial sign of chronic CNS inflammation such as cerebral vasculitis or autoimmune encephalitis [2].

Therefore, in this pilot study we investigated NfL and GFAP levels in blood from Post-COVID-19 patients with new daily persistent headache (n = 6, all female), defined as being different from previous primary headaches (if any), having started after the initial serological diagnosis of SARS-CoV-2 infection and persisting longer than 12 weeks. The quality of Post-COVID-19 headaches was described as a pounding or squeezing sensation, and the intensity was described as fluctuating between medium-intensity and high-intensity. These patients had been classified as mild during acute infections according to the WHO definition, i.e. they did not require high flow oxygen therapy or ventilation. In comparison, we also analyzed blood NfL levels in male and female patients diagnosed with mild COVID-19 (n = 17), severe COVID-19 (n = 11), and COVID-19-seronegative control subjects (n = 14). Specimen were obtained 14 ± 24 weeks after the initial diagnosis in mild and 8 ± 19 weeks in severe COVID-19 patients, and 33 ± 17 weeks in Post-COVID-19 headache patients.

GFAP levels were analyzed in all patients with Post-COVID-19 headache, but were only available in n = 8 patients with mild COVID-19, n = 4 severe COVID-19, and n = 8 COVID-19-negative controls.

All patient characteristics are described in Table 1. All measurements were performed on a SIMOA analyzer (Quanterix) using the corresponding SIMOA assay kits.

**Table 1:** Patient characteristics

Group	Patient #	Primary symptom/diagnosis	Secondary diagnosis	Weeks since COVID-19 infection	Age (years)	Gender	Neurological exam	cMRI	CSF
Persistent Post-COVID-19 headache	1	Headache	Migraine	32	49	Female	Normal	n.a	No inflammatory change
	2	Headache	Recurrent syncopes	16	23	Female	Normal	Normal	No inflammatory change
	3	Headache	None	52	46	Female	Normal	Normal	No inflammatory change
	4	Headache	Bechterew’s disease	23	36	Female	Fasciculations right M. vastus med	Normal	No inflammatory change
	5	Headache	Migraine	55	49	Female	Normal	Normal	No inflammatory change
	6	Headache	None	21	51	Female	Normal	n.a	No inflammatory change
Mild COVID-19	7	Encephalopathy	Chronic heart disease, CAD, hyper-cholesterinemia, asthma	60	64	Male	Delirium, reduced vigilance, attention deficit, confusion, agitation	Normal	No inflammatory change
	8	Impaired gait	Hyperthyreosis, reflux	0	64	Male	Sensory ataxia	Microangio-pathy	No inflammatory change
	9	Encephalopathy	Pneumonia, carotid stenosis	2.5	74	Female	Somnolence, increased muscle tone	Normal	No inflammatory change
	10	Dyspnea	Hypothyreosis, hypertension	1	47	Female	Normal	n.a	n.a
	11	Dyspnea	AV block °I	10	60	Male	Normal	n.a	n.a
	12	Cough	Diabetes type II, nephropathy, hypertension, dementia	3	86	Female	Normal	n.a	n.a
	13	Dyspnea	None	1	42	Female	Normal	n.a	n.a
	14	Dyspnea	COPD, CAD, renal insufficiency	3	63	Female	n.a	n.a	n.a
	15	Cold symptoms	Liver transplant, hepatitis C, renal insufficiency, osteoporosis, Diabetes type II	0	51	Male	Normal	n.a	n.a
	16	Fever	Agammaglobuliemia	1	29	Male	Normal	n.a	n.a
	17	Perineuritis n. optici	None	4	33	Male	Reduced vision	Perineuritis nervi optici	No inflammatory change
	18	Impaired vision	Allergic asthma	62	42	Female	Normal	Normal	No inflammatory change
	19	Impaired gait	None	65	75	Female	Sensory ataxia	n.a	n.a
	20	PNP	Hypertension	17	52	Female	Sensory ataxia	n.a	n.a
	21	Impaired gait	Asthma, cutaneous t-cell lymphoma, acantholytic dermatosis	2	60	Male	Sensory ataxia	n.a	n.a
	22	Cold symptoms	None	2	31	Female	n.a	n.a	n.a
	23	Cold symptoms	COPD	2.5	65	Male	Normal	n.a	n.a
Severe COVID-19	24	Cold symptoms	Prostate cancer	1	65	Male	Normal	n.a	n.a
	25	Dyspnea	Stroke, atrial fibrillation, hypertension,	1	84	Female	Different focal neurological deficits	n.a	n.a
	26	Syncope	Aortic aneurysm, sleep apnea, aortic valve replacement, hypercholesterolemia	1	53	Male	Horner syndrome right side, sensory deficit left lower arm	n.a	n.a
	27	Dyspnea	Renal insufficiency, CAD, peripheral artery disease, atrial fibrillation, prostate cancer, hyperlipidemia, diabetes type II	1	84	Male	Reduced vigilance	n.a	n.a
	28	Dyspnea	Autoimmune hepatitis, hypothyreosis, liver fibrosis, esophageal varices	0	54	Female	normal	n.a	n.a
	29	Cold symptoms	CAD, hypertension, diabetes type II	1	82	Female	General weakness	n.a	n.a
	30	Dyspnea	Cardiomoypathy, CLL, hypercholesteremia, cardiomyopathy, mitral regurgitation °I	2	82	Female	Normal	n.a	n.a
	31	Dyspnea	Perimyocarditis, atrial fibrillation, critical illness myopathy	64	68	Female	Hypesthesia	n.a	n.a
	32	Dyspnea	Autoimmune hepatitis, Hashimoto Thyroiditis, hypertension, COPD, critical illness myopathy	4	64	Female	Sensory ataxia	n.a	n.a
	33	Cold symptoms	Hypertension, diabetes type II, restless legs syndrome, hypothyreosis	3	76	Female	n.a	n.a	n.a
	34	Dyspnea	Heart Failure, atrial fibrillation, hypertension, Diabetes mellitus II, CAD, hyperlipidemia	4	77	Female	External oculo-motor nerve palsy	n.a	No inflammatory change
COVID-19-negative controls	35	PPA	Gonarthrosis, hypercholesterinemia, hyperhomocysteinemia, glaucoma	0	62	Female	Cognitive deficits	Temporal lobe atrophy	No inflammatory change
	36	Idiopathic intracranial hypertension	Asthma, thyroid carcinoma, sarcoidosis	0	42	Female	Normal	n.a	No inflammatory change
	37	Idiopathic intracranial hypertension	None	0	32	Male	Normal	n.a	n.a
	38	Headache	None	0	39	Male	Normal	n.a	n.a
	39	None	None	0	27	Female	Normal	n.a	n.a
	40	None	Diabetes mellitus II	0	34	Female	Normal	n.a	n.a
	41	Polyneuropathy	obstructive sleep apnea, hypogonadotropic hypogonadism	0	57	Male	Sensory ataxia	n.a	n.a
	42	Seizure	Structural epilepsy, asthma, Spondylose deformans, hypertension	0	73	Male	Reduced vigilance	n.a	n.a
	43	n.a	n.a	n.a	n.a	n.a	n.a	n.a	n.a
	44	n.a	n.a	n.a	n.a	n.a	n.a	n.a	n.a
	45	n.a	n.a	n.a	n.a	n.a	n.a	n.a	n.a
	46	n.a	n.a	n.a	n.a	n.a	n.a	n.a	n.a
	47	n.a	Hemophilia A, liver transplant, polyneuropathy, renal insufficiency	0	68	Male	n.a	n.a	n.a
	48	Cold symptoms	None	0	37	Female	Normal	n.a	n.a

Some clinical data were not available due to ethics proposal restrictions

*COPD* chronic obstructive pulmonary disease, *cMRI* cerebral magnetic resonance imaging, *CSF* cerebrospinal fluid, *CAD* coronary artery disease, *CLL* chronic lymphocytic leukemia, *PPA* primary progressive aphasia, *n.a.* not applicable

We found that NfL levels were similar in patients with persistent Post-COVID-19 headache, mild COVID-19 and COVID-19-seronegative controls, but significantly elevated in severe COVID-19 compared to patients with persistent Post-COVID-19 headache (Fig. 1A). Similarly, GFAP levels were comparable in patients with persistent Post-COVID-19 headache, mild COVID-19 and COVID-19-seronegative controls, but significantly elevated in severe COVID-19 compared to persistent Post-COVID-19 headache patients (Fig. 1B).

**Fig. 1:**
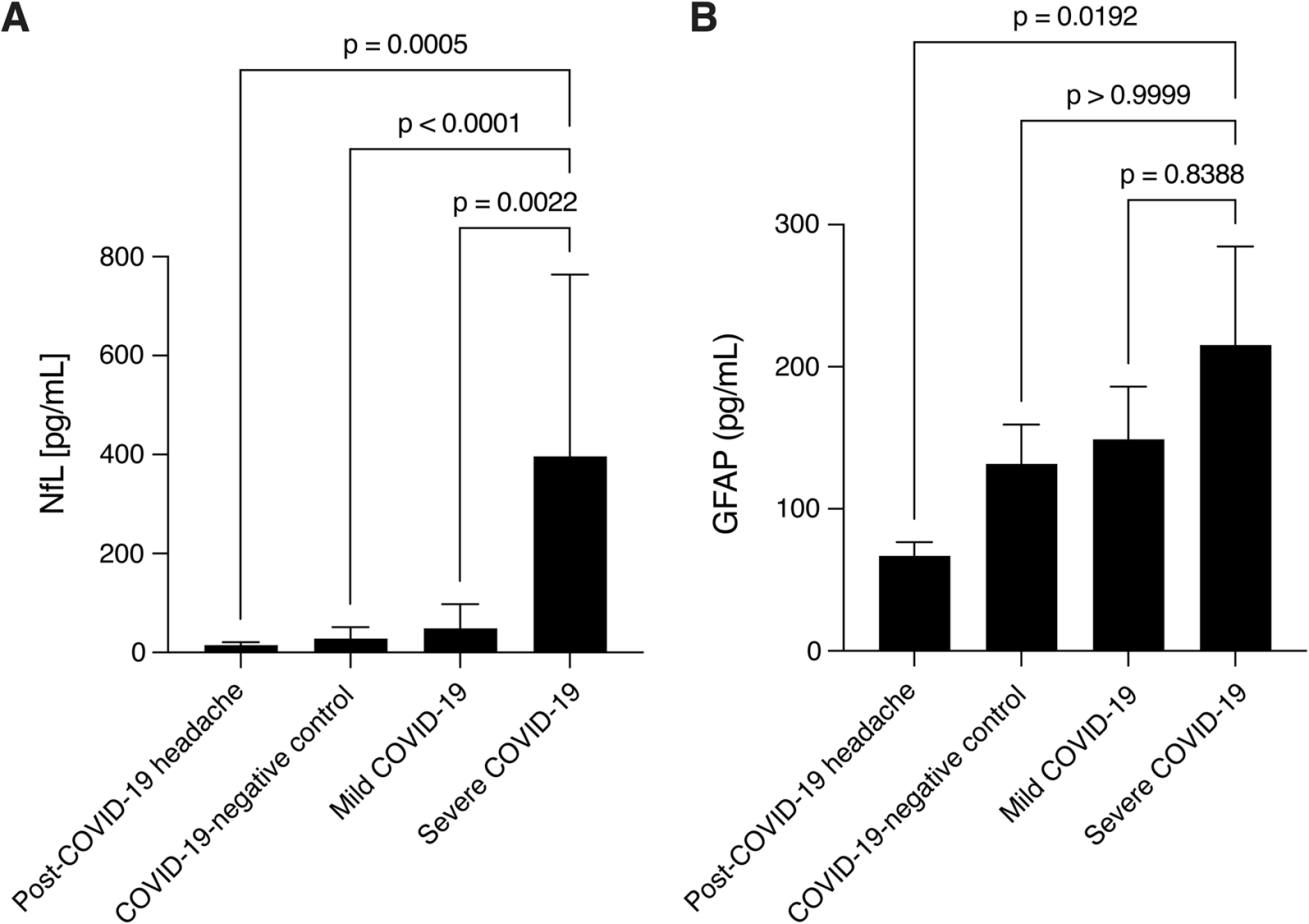
NfL and GFAP levels. **A** NfL levels in severe COVID-19 (n = 11) are significantly higher compared to persistent Post-COVID-19 headache (n = 6), mild COVID-19 (n = 17) and COVID-19-negative controls (n = 14; Kruskal–Wallis test followed by Dunn’s multiple comparisons test). **B** GFAP levels in persistent Post-COVID-19 headache (n = 6) were similar to compared to mild COVID-19 (n = 8) and COVID-19-negative controls (n = 8), but significantly lower compared to severe COVID-19 (n = 4; Kruskal–Wallis test followed by Dunn’s multiple comparisons test)

Thus, in contrast to severe COVID-19, we did not detect serological signs of CNS damage or reactive astrogliosis in patients presenting with persistent headache after mild COVID-19. Therefore, our data argue against persistent headache as an indicator of ongoing or progressive parenchymal damage or neuroinflammation. Moreover, our study suggests that persistent post-COVID-19 headache may be pathophysiologically and prognostically different from headache during acute COVID-19, which is often associated with elevated NFL and GFAP levels and may indicate increased mortality [1]. On the other hand, our data indicate that patients with severe COVID-19, even without neurological manifestations, should be closely monitored for ongoing CNS damage as this subgroup exhibited increased NfL and GFAP levels even after the acute phase of COVID-19. Limitations of this pilot study include the small sample sizes, missing follow-up analyses and clinical heterogeneity of groups. However, our study supports recent analyses that reported normal levels of CNS biomarkers in blood from COVID-19 patients with ongoing neurological symptoms [1, 4].

## Data Availability

The datasets used and analysed during the current study are available from the corresponding author on reasonable request.
